# Supplementary material for: Feasibility of establishing a serosurveillance system using residual specimens from district-level hospitals: A case study in Choma and Ndola Districts of Zambia
Source: PLoS One. 2026 Aug 4;21(8):e0341389. doi: 10.1371/journal.pone.0341389 (PMC13436745; doi:10.1371/journal.pone.0341389)
Supplement: Supplementary file 1 [file pone.0341389.s001.docx]

| **Facilities** | **Variability in procedures** | | |
| --- | --- | --- | --- |
|  | **Identifying specimens** | **Processing specimens** | **Data abstraction & data entry** |
| **Choma General (pediatric and adult collection)** | Paper lab request forms received that day were manually separated by age group (pediatric vs. adult) using age or ward on form. If unable to determine age group, then specimen was excluded. Lab staff compiled a list of eligible specimens.  Lab staff scanned specimen tube racks to locate eligible specimens.  Adult specimens were systematically sampled. All eligible pediatric specimens were included due to small numbers.  For those eligible specimens identified or selected, lab staff confirmed specimens were not from patients processed the prior two weeks, not from the ART clinic, had sufficient volume, and no severe hemolysis. | Assigned study identification number to the final set of eligible specimens.    Centrifuged then aliquoted to two labeled cryovials and placed them in freezer boxes. Selected replacement if the specimen was severely hemolyzed following centrifugation and additional eligible specimens available. | Lab staff provided a form listing names of the patients whose residual specimens were identified and processed to the facility’s data staff.  Data staff located patient files corresponding to the specified individuals and brought them to the lab. Additional work needed to locate patient files for pediatric patients to abstract data on vaccination status. In some situations, this required repeated visits to the wards, registry, and pharmacy, and speaking with clinical staff to locate records.  Lab staff abstracted data from files to data collection form on tablet. |
| **ADCH (pediatric collection only)** | Specimen tubes were organized by age category by using the name or laboratory identification number on the tube to locate the age in the electronic system. Specimens that were missing age were randomly distributed between the categories.  Specimens were systematically sampled from each age category.  Similar procedures as described above were used to confirm final eligibility of sampled specimens. | No differences from above. | For outpatients, the data team searched in SmartCare using the patient hospital number to abstract all information necessary except vaccination status.  For data on vaccination status and all data for inpatients, the data team located patient information in the ward register.  Abstracted data was compiled by a data team then transmitted to the lab staff to enter in data collection form on a tablet. |
| **NTH (adult collection only)** | Lab staff systematically sampled from the set of all available specimen tubes, excluding those from the ART clinic.  Selected samples with severe hemolysis or low volume were replaced.  Only the selected specimens were checked for age eligibility by looking up the ID number on the tube in the lab information management system (Disa). Age ineligible specimens were replaced.  The final set of selected specimens was checked for potential duplicate residual specimens from the same patient. | No differences from above. | Data team identified the ward where each patient was seen then located patient information in ward registers using the patient number and name.  One data point (referring facility) was only available at the registration points, requiring a separate visit to those locations to locate the individuals in the register and abstract data.  Abstracted data was compiled by a data team then transmitted to the lab staff to enter in data collection form on tablet. |
