# Supplementary material for: Feasibility of establishing a serosurveillance system using residual specimens from district-level hospitals: A case study in Choma and Ndola Districts of Zambia
Source: PLoS One. 2026 Aug 4;21(8):e0341389. doi: 10.1371/journal.pone.0341389 (PMC13436745; doi:10.1371/journal.pone.0341389)
Supplement: Supplementary file 2 [file pone.0341389.s002.docx]

Patient Characteristics

Who are the patients using the facility? Provide description of facility and catchment area with respect to the below considerations. Obtain copies of readily available facility-level summaries where possible.

- Epidemiological characteristics of catchment area

- Age distribution of patients

- Rural vs. urban distribution of patients

- Geographic distribution of patients (district-wide or subset)

- Percent of patients coming from outside the district

- Facility specialty (if applicable)

- Top causes of morbidity for patients presenting at the facility

Laboratory characteristics (*take photos of laboratory set up and equipment if possible)*

Which laboratories in the facility receive specimens from the target population? Describe the laboratories in terms of tests performed (e.g., hematology, biochemistry, molecular, microbiology) and which ward(s) they serve.

Are there any tests performed in the lab that require a smaller volume (<0.5mL) of blood (i.e., specimens less likely to have residual volume remaining)?

Describe the flow of specimens and data from the point of collection to the laboratory.

How long are specimens typically stored at the laboratory after testing before being discarded (range of days)? Would residual specimens be available immediately after testing or is there a period when they are stored in case of repeat testing?

How are the specimens stored during this period (bench-top, 2-8 C, etc.)?

Does the laboratory have a centrifuge and space the project staff can use to centrifuge and aliquot the residual specimens?

Does the laboratory have other equipment available?

- 2-8 C refrigerator (for short-term storage of whole blood)
- -20 C freezer (for longer term storage of sera specimens)

Data availability for residual specimens

Are the following data available for each specimen? *(check all sources of data that apply; provide additional details in space)*

*Obtain a copy of the lab request form and describe column headers or fields in the registers.*

- Age or date of birth *(on tube, in lab register, or in hospital register only)*

- Date of collection *(on tube, in lab register, or in hospital register only)*

- Gender *(on tube, in lab register, or in hospital register only)*

- Source of specimen (OPD vs. inpatient ward; hematology vs. biochemistry lab) *(on tube, in lab register, or in hospital register only)*

- Reason for blood collection (provisional diagnosis, tests ordered) *(in lab register, or in hospital register only)*

- Place of residence (e.g., town or village; to determine if individual lives in or out of district) *(in lab register, or in hospital register only)*

- Vaccination history *(in lab register, or in hospital register only, other specify)*

Format of lab register *(paper and/or electronic)*

Format of hospital register *(paper and/or electronic)*

Are there data points from the above list that seem to be missing for many patients? If a data point was missing, is there another source or way of obtaining that data?

Is there someone at the laboratory responsible for checking all lab forms to ensure completeness and quality? Describe any data quality control processes.

Would project staff be able to access information available in a hospital register (e.g., place and residence)? Is this data linked to the specimens (by name, by hospital ID, etc.)? *(if applicable)*

What is the easiest way for project staff to access the data (e.g., via printouts from the laboratory staff, accessing the computer database, transcribing from specimen tube or paper register)? *(aiming for limited effort on both the part of the laboratory staff and staff involved in residual specimen collection)*
