# Supplementary material for: Feasibility of establishing a serosurveillance system using residual specimens from district-level hospitals: A case study in Choma and Ndola Districts of Zambia
Source: PLoS One. 2026 Aug 4;21(8):e0341389. doi: 10.1371/journal.pone.0341389 (PMC13436745; doi:10.1371/journal.pone.0341389)
Supplement: Supplementary file 3 [file pone.0341389.s003.docx]

**Data point**

Hospital ward (inpatient/outpatient/not available)

Type of outpatient (Choma General only; based on health insurance status) ^a^

Medical department (surgical, medical, OB/GYN/ other, unknown)

Issues with clinical records (not located, damaged or illegible, incomplete, no problem)

If patient lives in district^a^

Health facility catchment area where patient resides based on clinical record^a^

Province and district, if residing outside district where health facility is located^a^

Age (calculated based on date of birth^b^)

Sex (male, female, not indicated)

Evidence of MCV receipt (documented, evidence one or more doses of MCV had not been received based on medical record, no mention of vaccination in record) ^a^

Date specimen collected from patient

Time specimen received at laboratory

Date residual specimen collected from laboratory

Volume

Hemolysis

Freezer storage box and position
