# Supplementary material for: Feasibility of establishing a serosurveillance system using residual specimens from district-level hospitals: A case study in Choma and Ndola Districts of Zambia
Source: PLoS One. 2026 Aug 4;21(8):e0341389. doi: 10.1371/journal.pone.0341389 (PMC13436745; doi:10.1371/journal.pone.0341389)
Supplement: Supplementary file 4 [file pone.0341389.s004.docx]

| **Category** | **Activities requiring resources** |
| --- | --- |
| Personnel | Identification, selection, and processing of residual specimens at health facility |
|  | Data abstraction at health facility |
|  | Local oversight and management at health facility |
|  | External coordination |
| Transportation | Transport of consumables to health facility |
|  | Transport of specimens from health facility to central laboratory for testing |
|  | Transport of coordination team to health facility for training, oversight, and engagement |
| Supplies | Specimen processing at health facility (e.g., microtainer tubes, filter tips) |
|  | Specimen testing at central laboratory (e.g., filter tips, tubes, assays) |
|  | Specimen packaging and transport (e.g., freezer boxes, cool packs) |
| Equipment | Specimen processing at health facility (e.g., centrifuge) |
|  | Specimen storage at health facility (-20°C or -80°C freezer) |
|  | Specimen storage at central laboratory (-20°C or -80°C freezer) |
|  | Computer or tablet for data abstraction |
