# Supplementary material for: Feasibility of establishing a serosurveillance system using residual specimens from district-level hospitals: A case study in Choma and Ndola Districts of Zambia
Source: PLoS One. 2026 Aug 4;21(8):e0341389. doi: 10.1371/journal.pone.0341389 (PMC13436745; doi:10.1371/journal.pone.0341389)
Supplement: Supplementary file 5 [file pone.0341389.s006.docx]

PONE-D-25-62563

Feasibility of establishing a serosurveillance system using residual specimens from district-level hospitals: A case study in Choma and Ndola Districts of Zambia

PLOS One

Dear Dr. Mutembo,

Thank you for submitting your manuscript to PLOS ONE. After careful consideration, we feel that it has merit but does not fully meet PLOS ONE’s publication criteria as it currently stands. Therefore, we invite you to submit a revised version of the manuscript that addresses the points raised during the review process.

Specifically, as stated by the first reviewer if the manuscript concludes that the system is feasible, but it would be stronger to provide a few simple quantitative indicators to support the conclusion. A short chapter untitled lesson learned migh help to highlight the key point of a successfull study using this "used remnent samples".

**Response**: We have revised the manuscript in many parts to address the feasibility of integrating residual specimen collection in routine hospital work. To do this we have revised table 1 to include part B and created a new table (Table 3) which has feasibility indicators. We have created a new section in the results section describing these indicators and towards the end of the manuscript, we highlight cross cutting lessons on facilitators and challenges. We have also revised the conclusion to align with the new revisions. You will find the detailed specific responses to the suggested revision below as we respond to each point.

Please submit your revised manuscript by Mar 22 2026 11:59PM. If you will need more time than this to complete your revisions, please reply to this message or contact the journal office at [plosone@plos.org](mailto:plosone@plos.org). When you're ready to submit your revision, log on to <https://www.editorialmanager.com/pone/>and select the 'Submissions Needing Revision' folder to locate your manuscript file.

Please include the following items when submitting your revised manuscript:

- A letter that responds to each point raised by the academic editor and reviewer(s). You should upload this letter as a separate file labeled 'Response to Reviewers'.
- A marked-up copy of your manuscript that highlights changes made to the original version. You should upload this as a separate file labeled 'Revised Manuscript with Track Changes'.
- An unmarked version of your revised paper without tracked changes. You should upload this as a separate file labeled 'Manuscript'.

If you would like to make changes to your financial disclosure, please include your updated statement in your cover letter. Guidelines for resubmitting your figure files are available below the reviewer comments at the end of this letter.

**Response**: No changes to financial disclosure and cover letter is attached summarizing all responses.

If applicable, we recommend that you deposit your laboratory protocols in protocols.io to enhance the reproducibility of your results. Protocols.io assigns your protocol its own identifier (DOI) so that it can be cited independently in the future. For instructions see: [https://journals.plos.org/plosone/s/submission-guidelines#loc-laboratory-protocols](https://nam02.safelinks.protection.outlook.com/?url=https%3A%2F%2Ftrack.editorialmanager.com%2FCL0%2Fhttps%3A%252F%252Fjournals.plos.org%252Fplosone%252Fs%252Fsubmission-guidelines%2523loc-laboratory-protocols%2F1%2F010f019c30ce1e26-d0454ed8-49eb-41c3-956e-cb84bc0f494b-000000%2FdcG-KVbxiG_nTBmtRj_0_sRRfanjBUg9EzD78LGpPec%3D249&data=05%7C02%7Csmutemb1%40jhmi.edu%7C9eb6eb5fcedb4675c7bf08de65286c69%7C9fa4f438b1e6473b803f86f8aedf0dec%7C0%7C0%7C639059421735728186%7CUnknown%7CTWFpbGZsb3d8eyJFbXB0eU1hcGkiOnRydWUsIlYiOiIwLjAuMDAwMCIsIlAiOiJXaW4zMiIsIkFOIjoiTWFpbCIsIldUIjoyfQ%3D%3D%7C0%7C%7C%7C&sdata=wSpkM9DJ%2FfTR8MeqG2ZUdmjkmaRPToD7FgNwj9Dkcgg%3D&reserved=0). Additionally, PLOS ONE offers an option for publishing peer-reviewed Lab Protocol articles, which describe protocols hosted on protocols.io. Read more information on sharing protocols at [https://plos.org/protocols?utm_medium=editorial-email&utm_source=authorletters&utm_campaign=protocols](https://nam02.safelinks.protection.outlook.com/?url=https%3A%2F%2Ftrack.editorialmanager.com%2FCL0%2Fhttps%3A%252F%252Fplos.org%252Fprotocols%253Futm_medium%3Deditorial-email%2526utm_source%3Dauthorletters%2526utm_campaign%3Dprotocols%2F1%2F010f019c30ce1e26-d0454ed8-49eb-41c3-956e-cb84bc0f494b-000000%2FtJDFuE01PF_h1VjPXNr8mj57jMMya4e-Qw979PKwuts%3D249&data=05%7C02%7Csmutemb1%40jhmi.edu%7C9eb6eb5fcedb4675c7bf08de65286c69%7C9fa4f438b1e6473b803f86f8aedf0dec%7C0%7C0%7C639059421735773110%7CUnknown%7CTWFpbGZsb3d8eyJFbXB0eU1hcGkiOnRydWUsIlYiOiIwLjAuMDAwMCIsIlAiOiJXaW4zMiIsIkFOIjoiTWFpbCIsIldUIjoyfQ%3D%3D%7C0%7C%7C%7C&sdata=LWEfxBdvJskqGiZKFYLvXBv%2BXcYGR83Hjt4lKym8Y6E%3D&reserved=0).

We look forward to receiving your revised manuscript.

Kind regards,

Pierre Roques, Ph.D.
Academic Editor
PLOS One

**Journal Requirements:**

When submitting your revision, we need you to address these additional requirements.

1. Please ensure that your manuscript meets PLOS ONE's style requirements, including those for file naming. The PLOS ONE style templates can be found at
   <https://journals.plos.org/plosone/s/file?id=wjVg/PLOSOne_formatting_sample_main_body.pdf> and 
   <https://journals.plos.org/plosone/s/file?id=ba62/PLOSOne_formatting_sample_title_authors_affiliations.pdf>

**Response**: The manuscript is formatted according to PLOS ONE style.

2. In the online submission form you indicate that your data is not available for proprietary reasons and have provided a contact point for accessing this data. Please note that your current contact point is a co-author on this manuscript. According to our Data Policy, the contact point must not be an author on the manuscript and must be an institutional contact, ideally not an individual. Please revise your data statement to a non-author institutional point of contact, such as a data access or ethics committee, and send this to us via return email. Please also include contact information for the third-party organization, and please include the full citation of where the data can be found.

**Response:** We have consulted the Zambia National Health Research Authority. Anonymized data and code will be upload to the Github link. <https://github.com/HopkinsIVAC/ResidualFeasibility>

3. When completing the data availability statement of the submission form, you indicated that you will make your data available on acceptance. We strongly recommend all authors decide on a data sharing plan before acceptance, as the process can be lengthy and hold up publication timelines. Please note that, though access restrictions are acceptable now, your entire data will need to be made freely accessible if your manuscript is accepted for publication. This policy applies to all data except where public deposition would breach compliance with the protocol approved by your research ethics board. If you are unable to adhere to our open data policy, please kindly revise your statement to explain your reasoning and we will seek the editor's input on an exemption. Please be assured that, once you have provided your new statement, the assessment of your exemption will not hold up the peer review process.

**Response**: We have uploaded the data to following institutional website:

<https://github.com/HopkinsIVAC/ResidualFeasibility>

4. PLOS requires an ORCID iD for the corresponding author in Editorial Manager on papers submitted after December 6th, 2016. Please ensure that you have an ORCID iD and that it is validated in Editorial Manager. To do this, go to ‘Update my Information’ (in the upper left-hand corner of the main menu), and click on the Fetch/Validate link next to the ORCID field. This will take you to the ORCID site and allow you to create a new iD or authenticate a pre-existing iD in Editorial Manager.

**Response**: https://orcid.org/0000-0002-5389-5966

5. We note that you have included the phrase “data not shown” in your manuscript. Unfortunately, this does not meet our data sharing requirements. PLOS does not permit references to inaccessible data. We require that authors provide all relevant data within the paper, Supporting Information files, or in an acceptable, public repository. Please add a citation to support this phrase or upload the data that corresponds with these findings to a stable repository (such as Figshare or Dryad) and provide and URLs, DOIs, or accession numbers that may be used to access these data. Or, if the data are not a core part of the research being presented in your study, we ask that you remove the phrase that refers to these data.

**Response**: As above: Here is the link

<https://github.com/HopkinsIVAC/ResidualFeasibility>

And revised the manuscript and other submission portal sections accordingly.

6. Please include your full ethics statement in the ‘Methods’ section of your manuscript file. In your statement, please include the full name of the IRB or ethics committee who approved or waived your study, as well as whether or not you obtained informed written or verbal consent. If consent was waived for your study, please include this information in your statement as well.

**Response**: Revised ethics statement line 100 – 105:

“Ethical approval for this study was obtained from the Johns Hopkins Bloomberg School of Public Health Institutional Review Board, the National Health Research and Training Institute Research Ethics Committee, and the Zambia National Health Research Authority. Letters of permission were provided by participating hospitals. All committees granted a waiver of informed consent because only deidentified residual specimens and deidentified metadata were used, with no patient contact or identifiable information collected.”

7. Please upload a new copy of Figure 1 as the detail is not clear. Please follow the link for more information:  <https://journals.plos.org/plosone/s/figures>

**Response:** We have revised figure 1

8. If the reviewer comments include a recommendation to cite specific previously published works, please review and evaluate these publications to determine whether they are relevant and should be cited. There is no requirement to cite these works unless the editor has indicated otherwise.

**Response**: N/A

[Note: HTML markup is below. Please do not edit.]

Reviewers' comments:

Reviewer's Responses to Questions

**Comments to the Author**

1. Is the manuscript technically sound, and do the data support the conclusions?

The manuscript must describe a technically sound piece of scientific research with data that supports the conclusions. Experiments must have been conducted rigorously, with appropriate controls, replication, and sample sizes. The conclusions must be drawn appropriately based on the data presented.

Reviewer #1: Partly

Reviewer #2: Yes

**Response**: We have revised parts of the manuscript to improve technical strength of the manuscript and included more data in response to peer reviewers’ suggestions. Please refer to specific point-point responses and the cover letter for these changes.

2. Has the statistical analysis been performed appropriately and rigorously?

Reviewer #1: N/A

Reviewer #2: N/A

**Response**: Thanks.

3. Have the authors made all data underlying the findings in their manuscript fully available?

The [PLOS Data policy](https://nam02.safelinks.protection.outlook.com/?url=http%3A%2F%2Ftrack.editorialmanager.com%2FCL0%2Fhttp%3A%252F%252Fwww.plosone.org%252Fstatic%252Fpolicies.action%2523sharing%2F1%2F010f019c30ce1e26-d0454ed8-49eb-41c3-956e-cb84bc0f494b-000000%2FfiAkdNrTDb5oa_k5-EfVpf5UM69Xc07BdRVwGN45QTc%3D249&data=05%7C02%7Csmutemb1%40jhmi.edu%7C9eb6eb5fcedb4675c7bf08de65286c69%7C9fa4f438b1e6473b803f86f8aedf0dec%7C0%7C0%7C639059421735795647%7CUnknown%7CTWFpbGZsb3d8eyJFbXB0eU1hcGkiOnRydWUsIlYiOiIwLjAuMDAwMCIsIlAiOiJXaW4zMiIsIkFOIjoiTWFpbCIsIldUIjoyfQ%3D%3D%7C0%7C%7C%7C&sdata=ZZxejKmWFvSjonf%2Fo8MohlASgIFzqxKEO4YzKPdPcf0%3D&reserved=0) requires authors to make all data underlying the findings described in their manuscript fully available without restriction, with rare exception (please refer to the Data Availability Statement in the manuscript PDF file). The data should be provided as part of the manuscript or its supporting information, or deposited to a public repository. For example, in addition to summary statistics, the data points behind means, medians and variance measures should be available. If there are restrictions on publicly sharing data—e.g. participant privacy or use of data from a third party—those must be specified.

Reviewer #1: Yes

Reviewer #2: No

**Response**: We have shared the link where the anonymized data and code is archived

4. Is the manuscript presented in an intelligible fashion and written in standard English?

PLOS ONE does not copyedit accepted manuscripts, so the language in submitted articles must be clear, correct, and unambiguous. Any typographical or grammatical errors should be corrected at revision, so please note any specific errors here.

Reviewer #1: Yes

Reviewer #2: Yes

**Response**: Thank you

5. Review Comments to the Author

Please use the space provided to explain your answers to the questions above. You may also include additional comments for the author, including concerns about dual publication, research ethics, or publication ethics. (Please upload your review as an attachment if it exceeds 20,000 characters)

Reviewer #1: Dear Editor,

Thank you for the opportunity to review this manuscript. The topic is important and the idea of using residual specimens for serosurveillance is interesting, especially in low-resource settings. The paper is clear and well written overall.

However, I have a few concerns that should be addressed before publication.

Feasibility outcomes: the manuscript concludes that the system is feasible, but it would be stronger to provide a few simple quantitative indicators (for example, number of eligible samples per day, proportion excluded, missing data rate for key variables, and basic information on workload/time). Even descriptive metrics by site would help.

**Response:** We appreciate the reviewer comment and realize this is critical point of the manuscript and have endevoured to revise the manuscript to improve clarity and provide more detail on the feasibility aspects. We have revised table 1 now referred to as “Health Facility characteristics and residual specimen collection parameters” to include some of these indicators (Part B: Residual specimen collection) and created an addition table (now Table 3 titled “Residual specimens identified and excluded per day and reasons for exclusion”) to present extra indicators that will address feasibility outcomes. These feasibility outcome indicators for the specimens processed and excluded were only available for one hospital.

We have also created a subsection in the result title : “Lessons learned integrating into routine hospital workflows”

Sampling strategy and duplicates: the daily “systematic sampling” approach should be described more precisely (how were samples selected during the day?). Also, the definition of duplicates (2-week window) is pragmatic but needs a brief justification, and ideally the number of duplicates excluded.

**Response:** We have included the number of duplicates excluded based on daily tracking data from one facility. Refer to Table 2 and added text in the manuscript to explicitly explain systematic sampling. “The systematic sampling interval varied based on the anticipated number of specimens received in the hematology laboratory per day based on the historical inventory and the target number of residual specimens to be collected per day. The sampling interval was reassessed periodically and adjusted as needed if the observed number of specimens received differed from the historical data or if the number of residual specimens collected per day needed to be increased or decreased.”

We have added the following sentence to the manuscript to qualify the 2-week window (Line 120):

“A two-week window was selected to reduce burden when searching for duplicates and assuming the patient’s serostatus may have changed outside that time frame due to exposure or vaccination.”

Data availability: the current statement (“available upon request”) may not fully match the journal data sharing policy. Please clarify what data can be shared and how (even a de-identified dataset or limited variables).

**Response**: We have uploaded the data set on the github. Here is the link: <https://github.com/HopkinsIVAC/ResidualFeasibility>

Minor comments:

Please check Table 1 layout to make totals easier to read.

**Response**: New revised and improved table 1 has been included in the manuscript. The revised table includes information to address the feasibility concerns raised by the reviewer

A short paragraph summarizing the main “lessons learned” (Table 2) in the Discussion could improve readability.

**Response:** We have included a section in the discussion highlighting the key lessons from table 2 and linking these lessons to previous literature and practical implications Line 441-455.

In summary, I think this work is valuable, but I recommend major revision to strengthen the feasibility assessment and improve clarity on sampling and data sharing.

**Response:** We have made revisions in the text and tables of the manuscript to respond to all these very important comments. In our revisions we have addressed the feasibility assessment, improved clarity on sampling and will upload our anonymized data set to the github link. As a result of these revisions we have also revised the conclusion to align with the revisions made to the manuscript text and tables.

Sincerely,

Reviewer #2: 1-OUI. Le manuscrit présente une étude de faisabilité bien conçue avec des objectifs clairs, des méthodes appropriées et une collecte systématique de données à travers trois hôpitaux. La combinaison d’observations directes, de cartographie des processus et de consultations structurées avec les parties prenantes est méthodologiquement solide et appliquée de manière cohérente. Les données rapportées (par exemple, le nombre d’échantillons dans le tableau 1, le flux de travail dans la Figure 1 et les leçons dans le tableau 2) soutiennent directement les principales conclusions selon lesquelles la collecte des échantillons résiduels est faisable mais nécessite des ajustements opérationnels pour la scalabilité. Les auteurs sont prudents dans l’interprétation de leurs conclusions, reconnaissent les limites, et évitent de surestimer la généralisation. Dans l’ensemble, l’étude est techniquement rigoureuse, et les conclusions sont bien étayées par les preuves fournies.

**Response**: We thank the reviewer for this very positive assessment. We are pleased that the combination of direct observations, process mapping, and structured stakeholder consultations was found to be rigorous and appropriately applied. No changes were required based on this comment.

2- N/A
Il s’agit d’une étude descriptive de faisabilité axée sur les flux de travail opérationnels et la collecte de spécimens dans trois hôpitaux. Aucune analyse statistique formelle n’est prévue ni requise, car le manuscrit ne vise pas à tester des hypothèses ni à estimer les tailles d’effet. Les résultats reposent sur des observations qualitatives, la cartographie des processus, des consultations avec le personnel et des décomptes sommaires simples (par exemple, les spécimens collectés par groupe d’âge et site dans le tableau 1). L’absence de statistiques inférentielles est appropriée pour ce type de recherche axée sur la mise en œuvre, et les données descriptives fournies suffisent à étayer les conclusions. Des contextes supplémentaires, tels que les proportions des échantillons exclus ou les distributions de temps de flux de travail, pourraient améliorer la clarté mais n’affectent pas la validité globale des résultats.

**Response**: We agree that inferential statistics are not necessary for this feasibility design. However, in response to comments from Reviewer #1, we have added additional descriptive feasibility indicators—such as number of eligible specimens per day, exclusion proportions, missing‑data rates, and duplicate counts—which also address the reviewer’s suggestion for enhanced contextual clarity. These additions strengthen the descriptive assessment without changing the study objectives.

3- NON ; Je recommande aux auteurs soit de fournir des tableaux résumés anonymisés ou des extraits dans les informations de soutien, soit de spécifier un point de contact indépendant pour un accès afin de mieux s’aligner sur la politique de la revue.

**Response:**
We appreciate this suggestion and have revised the Data Availability Statement accordingly. The updated statement clarifies that: a **fully anonymized dataset** containing key variables related to feasibility (e.g., specimen counts, eligibility indicators, and metadata availability) has been upload on the Github link: <https://github.com/HopkinsIVAC/ResidualFeasibility>

4- OUI ; Le manuscrit est clairement rédigé et bien structuré, suivant un flux logique. Les termes techniques sont utilisés de manière appropriée et cohérente, et le texte est entièrement intelligible pour un public international.

**Response:**
We thank the reviewer for this positive feedback. We have further refined several sections for clarity during the revision process, but no substantive changes were required to address this comment.

6. PLOS authors have the option to publish the peer review history of their article ([what does this mean?](https://nam02.safelinks.protection.outlook.com/?url=https%3A%2F%2Ftrack.editorialmanager.com%2FCL0%2Fhttps%3A%252F%252Fjournals.plos.org%252Fplosone%252Fs%252Feditorial-and-peer-review-process%2523loc-peer-review-history%2F1%2F010f019c30ce1e26-d0454ed8-49eb-41c3-956e-cb84bc0f494b-000000%2FS5t1VzY4K6gpSd_3BqnVzTAUrWnO2SNwMuOocS5laSs%3D249&data=05%7C02%7Csmutemb1%40jhmi.edu%7C9eb6eb5fcedb4675c7bf08de65286c69%7C9fa4f438b1e6473b803f86f8aedf0dec%7C0%7C0%7C639059421735815901%7CUnknown%7CTWFpbGZsb3d8eyJFbXB0eU1hcGkiOnRydWUsIlYiOiIwLjAuMDAwMCIsIlAiOiJXaW4zMiIsIkFOIjoiTWFpbCIsIldUIjoyfQ%3D%3D%7C0%7C%7C%7C&sdata=3DaJfAl5Xr2Op94JtJmXhh%2FndmP9a9PEkoYHA0oXvV0%3D&reserved=0)). If published, this will include your full peer review and any attached files.

If you choose “no”, your identity will remain anonymous but your review may still be made public.

**Do you want your identity to be public for this peer review?** For information about this choice, including consent withdrawal, please see our [Privacy Policy](https://nam02.safelinks.protection.outlook.com/?url=https%3A%2F%2Ftrack.editorialmanager.com%2FCL0%2Fhttps%3A%252F%252Fwww.plos.org%252Fprivacy-policy%2F1%2F010f019c30ce1e26-d0454ed8-49eb-41c3-956e-cb84bc0f494b-000000%2FJaaqzK6TF2i2jVhJ7b_zDTo-MjkTyGyc-fJXa93bPSA%3D249&data=05%7C02%7Csmutemb1%40jhmi.edu%7C9eb6eb5fcedb4675c7bf08de65286c69%7C9fa4f438b1e6473b803f86f8aedf0dec%7C0%7C0%7C639059421735836451%7CUnknown%7CTWFpbGZsb3d8eyJFbXB0eU1hcGkiOnRydWUsIlYiOiIwLjAuMDAwMCIsIlAiOiJXaW4zMiIsIkFOIjoiTWFpbCIsIldUIjoyfQ%3D%3D%7C0%7C%7C%7C&sdata=RVnLXcimvPobssyTsFhOPnvWd%2FJE%2BCoBw7pOEY0eHIA%3D&reserved=0).

Reviewer #1: No

Reviewer #2: No

[NOTE: If reviewer comments were submitted as an attachment file, they will be attached to this email and accessible via the submission site. Please log into your account, locate the manuscript record, and check for the action link "View Attachments". If this link does not appear, there are no attachment files.]

To ensure your figures meet our technical requirements, please review our figure guidelines: <https://journals.plos.org/plosone/s/figures>

You may also use PLOS’s free figure tool, NAAS, to help you prepare publication quality figures: <https://journals.plos.org/plosone/s/figures#loc-tools-for-figure-preparation>.

NAAS will assess whether your figures meet our technical requirements by comparing each figure against our figure specifications.
